# How can we best distinguish rapidly progressive from slowly progressive precocious puberty in girls?

**DOI:** 10.1210/jendso/bvag221

**Published:** 2026-09-26

**Authors:** Paul Kaplowitz

**Affiliations:** Division of Endocrinology, Children's National Hospital, Washington, DC 20010, USA

**Keywords:** precocious puberty, slowly progressive, obesity, DXA body fat distribution, luteinizing hormone (LH), bone age

The evaluation of girls with early breast development can be challenging, particularly because it is often not clear at the initial visit which girls have the rapidly progressive CPP (RP-PP) who might benefit from treatment vs those with premature thelarche (largely girls under age 3) or non- or slowly progressive PP who may not require treatment to achieve normal timing of menarche and adult height. Basal and GnRH-stimulated LH levels and BA advancement are sufficient in most cases to make this distinction, but the common presence of overweight and obesity in early maturing girls can make a diagnosis of CPP more challenging, in part because it may be difficult to distinguish breast from adipose tissue. Adiposity is clearly an important factor influencing the onset and progression of puberty, with many studies showing earlier thelarche and menarche in girls with higher BMI (1, 2), with the fat-derived protein leptin being implicated in this relationship. The authors of the study discussed here have previously looked at DEXA as a way of seeing if regional fat distribution might differ between girls with rapid progressive vs slowly progressive PP (3) and now expand their observations to see how adiposity may contribute to these differences.

They performed a retrospective observational study at a single center in Italy, reviewing the medical records of 100 girls aged 5-9 years who were referred for suspected CPP or early pubertal development and who underwent DXA as part of their evaluation between January 2022 and May 2024 (4). Sixty-nine girls met the hormonal criteria (including GnRH testing in most) for central precocious puberty (CPP, onset before age 8) or early puberty (EP, onset between ages 8 and 9) and were classified as CPP/EP. Thirty-one girls with breast development before age 9 were classified as non-progressive premature breast development (npPBD) after a 6-12-month observation period. All had a prepubertal gonadotropin profile, and non-progression was confirmed by stable or regressing breast Tanner stage. Overall, 44% of the sample were overweight or obese, with the npPBD girls having a greater percentage with obesity. There was no difference between height, weight, BMI and bone age between CPP/EP and npPBD girls. As previous work has shown, LH, FSH, estradiol, and also IGF-1 SDS helped distinguish between the two groups. Overall body composition was comparable, except that the girls with npPBD had a somewhat higher android-to-gynoid fat ratio (*P* = .03). Among normal-weight girls, those with CPP/EP showed a higher height SD (1.28) than those with npPBD (0.86), but for overweight/obese girls there was no difference (1.76 vs 1.82). Similarly for bone age advancement, it was greater in normal weight CPP girls than those with npPBD (1.34 vs 0.63 years), whereas in girls with overweight/obesity, there was no significant difference (1.82 vs 1.64 years). The authors conclude that overweight/obesity reduces the diagnostic specificity of the growth and bone age parameters in girls with early breast development and that measuring fat distribution using DXA did not help distinguish between CPP/EP and npPTD.

Two papers from 1999 and 2000 (5, 6) described a small number of girls (20 in 1 study and 17 out of 26 girls in the other) with early breast development who, after prolonged follow-up without treatment, showed only modest bone age advancement and achieved a normal adult height and a normal mean age at menarche of 11-12 years. Both authors pointed out that not all girls with early signs of puberty require treatment, but that careful follow-up is needed. The recently published Endocrine Society guidelines on central precocious puberty (7) addressed this question with 2 recommendations.

Recommendation 1: *“In girls who present with thelarche (Tanner B2) between ages 7.0 and 8.0 years, we suggest watchful waiting via periodic physical examinations rather than immediately performing evaluation with laboratory testing and/or radiologic imaging”*

Recommendation 2: *“In girls younger than age 7 years with initial breast development (Tanner B2), we suggest a 4-to 6-month period of observation to differentiate unsustained or slowly progressive puberty from rapidly progressive puberty before starting diagnostic evaluation.”*

A systematic review identified 11 case series published from 1992-2023 which reported on the slowly progressive variant of precocious puberty (7). Most studies were retrospective, and different studies used different definitions of slowly progressive PP (SP-PP) vs rapidly progressive PP (RP-PP), with the RP-PP girls generally showing advancement of at least 1 Tanner breast stage within 6 months. Several looked at factors which might be predictive of RP-PP including bone age advancement, serum LH and estradiol, and uterine and ovarian size and pelvic ultrasound. The proportion of patients with SP-PP in these studies ranged from 33-67%.

Perhaps the most useful study was that of Chen et al 2021 which prospectively followed 212 7-8-year-old girls from one center in China (8). They concluded that 55% of their sample had SP-PP and 45% had RP-PP. It was noted that 74 of 95 RP-PP girls had Tanner 3 breast development whereas only 2/99 SP-PP girls were Tanner 3. Girls with SP-PP were taller with a mean height SDS of 0.86 vs 0.03. An advanced bone age was the best predictor of a girl having RP-PP, with RP-PP girls having an average BA-CA of 1.48 years vs 0.48 years in the SP-PP girls.

The paper of Fava et al shows that obesity in girls with signs of early puberty makes it more difficult to distinguish between progressive vs non-progressive early puberty, because growth and bone age differences were diminished by obesity. It also adds the literature that many girls with early breast development will do fine with periodic follow-up and no treatment, since they carefully followed the subset of girls who were not treated to confirm the lack of rapid progression. Their use of fat distribution by DXA as a means of determining which girls were likely to progress may not have been successful. As other studies have reported, they found that advanced bone age and elevated pubertal hormones (as well as IGF-1 SD score) were the best predictors of rapid progression. However, as the new Endocrine Society guidelines propose, holding off on any testing at the initial visit and reassessing growth and pubertal progression in 4-6 months is a very viable strategy to diagnose RP-PP vs SP-PP for those with early Tanner 2 breast development and will avoid unnecessary testing and treatment in many girls.
